# Donald’s Ideotype and Growth Redundancy: A Pot Experimental Test Using an Old and a Modern Spring Wheat Cultivar

**DOI:** 10.1371/journal.pone.0070006

**Published:** 2013-07-25

**Authors:** Li Zhu, Da-Yong Zhang

**Affiliations:** State Key Laboratory of Earth Surface Processes and Resource Ecology and Ministry of Education Key Laboratory for Biodiversity Science and Ecological Engineering, Beijing Normal University, Beijing, China; University of Nottingham, United Kingdom

## Abstract

Human selection for high crop yield under water-limited conditions should have led modern cereal cultivars to invest less in root biomass, be it unconsciously. To test this hypothesis we conducted a pot experiment with two spring wheat cultivars, one old and one modern, both widely grown in the semi-arid regions of China. Using the replacement series method introduced by de Wit, we showed that the older landrace (Monkhead) was significantly more competitive than the more-modern cultivar (92-46). However, when grown in pure stand, old Monkhead had grown root biomass 3.5 times modern 92-46, whereas modern 92-46 gained a 20% higher grain yield. We also found modern 92-46 significantly increased root biomass per plant and root allocation (i.e., root biomass/total individual biomass) as its frequency in mixtures decreased, whereas old Monkhead did not respond in a similar way. This result suggests that the roots of modern cultivars may have gained an ability to recognize neighboring root systems and show more plastic self-restraining response to intra-cultivar competition.

## Introduction

Although one may quibble about the exact numbers, some population growth is generally considered inevitable, which indicates that global crop production will have to increase by at least 70% by the year 2050 [1]. Dealing with this challenge will require a combination of approaches, among which raising crop yield on existing cropland area continues to be a top priority over the next several decades.

For most plant breeders, the primary objective of breeding has been to boost crop yield all the time [2]. However, it has not been widely recognized that they should practice ‘group selection’ to maximize the collective performance of crops, because there could be a tradeoff between individual grain production and grain production by the whole crop [3,4,5,6]. It is true that the most competitive plants in a crop will gain a disproportionate share of the limiting resource in the environment, allowing them to produce more seeds and hence being ‘naturally selected’. But as they increased in frequency, they started competing mainly with each other. In such cases, there will be no longer any advantage to the strong competitors simply because their fellow neighbors are equally competitive. Because they still paid the higher cost of excessive investment in resource foraging structures (e.g., root or shoot), they actually produced fewer seeds per plant than the less competitive plants they displaced.

Donald [4] first noted the ubiquity of the tradeoff between individual competitiveness and group productivity and then argued that high yielding crop plants should be weak competitors, in opposition to what past natural selection would have brought about. The ‘weak competitor’ is a key point to the ideotype theory of Donald, but seems to have caused some semantic confusion in the literature. Zhang et al. [5] instead suggested the use of another term, ‘growth redundancy’, to make it immediately transparent that the “redundant” growth in resource-foraging structures could be sacrificed for increased yields. Using a game theoretical model, Zhang et al. [5] were able to demonstrate that maximizing grain yield in the population is not an evolutionary stable strategy, as the population can be invaded by mutant genotypes with higher competitive ability. It should be noted that root allocation may be modeled either as a genetically fixed traits (as in [5]) or as a plastic response (as in some game-theoretical models and the associated experimental tests [7,8]). Failure to note this difference has brought about some unfortunate confusion in the literature [8].

Zhang et al. [5] also linked Donald’s ideotype or growth redundancy to the famous ‘tragedy of the commons’ [9]: every member of a crop could do better if they all agree to invest less in aggressive competition, but unilateral restraint will be exploited. This may sound bad for plants engaged in competition, but precisely good news for agriculture since there may have left plenty of room to enhance crop yield per unit area through plant breeding [3,4,5,6]. Indeed, we have already seen some reversal of past natural selection in certain situations. The most notable example of this kind is the marked reduction in height during the green revolution. Shorter but higher-yielding plants are undoubtedly weak competitors in a light-limited crop, providing much-needed empirical evidence for the Donald’s view, but it was rarely seen this way [3]. For example, it had been argued that the traits identified by Donald [4] for developing weakly competitive plants simply reflected the trend towards changed crop architecture already established by plant breeders during the green revolution [10,11]. However, trade-offs between individual competitiveness and yield could also occur below-ground when water is limiting, and lower investment in roots is consistent with Donald’s idea while not subject to alternative explanations. Nevertheless it needs to be tested empirically whether modern cultivars moves towards weaker competitive ability, and if this is indeed the case, whether this effect is linked to a reduced root biomass.

Previous studies had found that old crop cultivars did possess a larger root system than modern cultivars [12,13,14,15]. However, most of those studies were conducted in field conditions, and accurate quantification of root growth for individual plants was mostly impossible. Also, little was done to directly evaluate relative competitive ability of old and modern cultivars. A field research we did before [16] showed that the local landrace of spring wheat, Monkhead, was more competitive, but lower yielding, than a modern cultivar. However, the root behavior was not monitored because it was difficult to excavate soil and sort roots out under field conditions. In this research, we conducted a pot experiment of competition between the local landrace Monkhead and a modern cultivar 92-46, which was designed in such a way that the root growth of each cultivar can be measured separately. As a result, we could establish a direct link between competitive ability and root growth. To assess the outcome of competition we used the de Wit replacement series method [17], in which the two competing cultivars are maintained at one constant overall density while the proportions of the two cultivars are changed. Our experimental design also enables us to determine how roots of each cultivar respond to differential proportions of neighboring root systems. In this regard, it is surprising to find that root system growth of the modern cultivar is more plastic and more influenced by the surrounding environment than that of the old cultivar.

## Materials and Methods

### Materials

Two spring wheat (*Triticum aestivum* L.) cultivars, old Monkhead and modern 92-46, were used in our experiment. Monkhead was the local landrace in semi-arid regions of Gansu Province, China, which was widely grown during the period of time from 1940 to 1970. Monkhead was a typical awnless and highly-tillering cultivar, whereas modern 92-46 was released in 2000 and still widely grown in the area. The two cultivars have similar phenologies, but 92-46 has awns and thus could be easily distinguished from Monkhead at harvest.

### Experimental design

**Figure 1:**
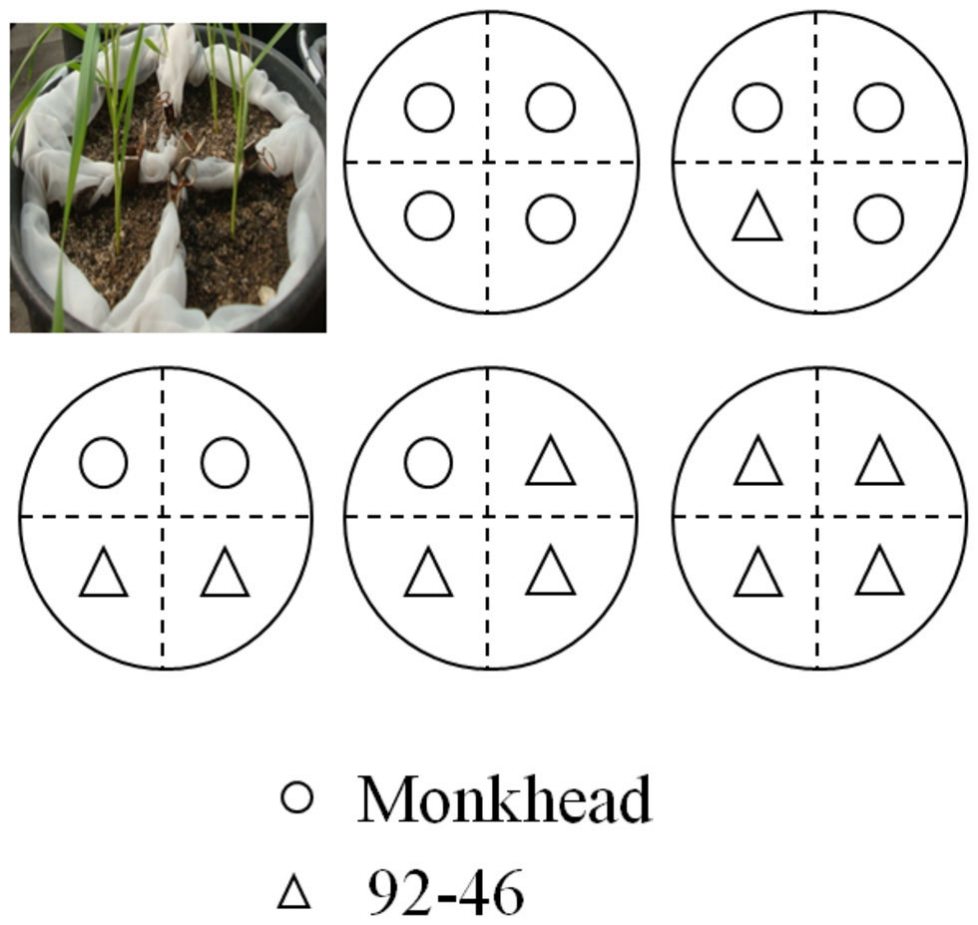
De Wit replacement series. 92-46 and Monkhead were mixed in ratios of 0: 4 (pure Monkhead), 1:3, 2:2, 3:1 and 4: 0 (pure 92-46), with four plants in each pot. The dotted line represented segregation by nylon bags.

We conducted a pot experiment in the botanic garden of Beijing Normal University (116° 2´E, 39° 6´N, ~ 54 m a.s.l.) from April to July in 2009, using de Wit replacement series method [17]. Each pot (25 cm diameter × 38 cm height) was drilled 3 holes in the bottom to let extra water out, and contained four individual plants as shown in Figure 1. Experiment was replicated 20 times, with mixture ratios (92-46: Monkhead) of 0: 4, 1:3, 2:2, 3:1, and 4: 0.

Growth substrate was a mixture of peat soil and sand with a mass ratio of 3:2. We filled the substrate into each pot to the same level below the edge of the pot. Substrate in each pot was separated and enclosed into four nylon bags (the pore size: 150 µm), which prevented plants from growing roots into their neighborhood but allowed water and nutrients to freely exchange between them. All seeds were vernalized at 4 ^°^C for 10 h and three seeds of the same cultivar were sown in 5-7 April in the central position of each bag. Two weeks after germination, seedlings were thinned to one for each bag. Pots were initially placed in the greenhouse of Beijing Normal University and then moved out in 13 May until harvest. They were arranged into 4 groups of 5×5, and placed one by one within each group. Such an arrangement of pots allowed plants access to more light than at field spacing, because within-group plant density per unit area was merely about one-tenth of the latter.

At maturity, both the aboveground parts and the roots were measured for each cultivar within each pot. Roots were separated from the substrate and cleaned with tap water. Above parts and roots were dried at 65 ^°^C for 5 days and weighed. Seeds of each plant peeled out from ears were counted and weighed.

### Statistical analysis

To compare the competitiveness of Monkhead and 92-46, we examined both seed biomass and seed number. We used *t*-test to compare seed biomass per pot of the two cultivars in mixture 2:2. We used seed number of each cultivar in three mixture treatments to make an input–output ratio diagram [18]. The output proportion of 92-46 at harvest is plotted against the input proportion of 92-46 at sowing. We used Wilcoxon Signed-rank test to examine if three output proportion values were significantly different from what is expected under the null hypothesis of equal competitive ability, i.e., the respective input proportions.

To compare the performance of the two cultivars in monoculture, we examined root biomass, seed biomass, and total biomass per plant (averaged from four plants per pot, respectively) using *t*-test analysis. We also examined root biomass, seed biomass and total biomass per plant along the de Wit replacement series using ANOVA, with biomass as dependent variable and de Wit series as fixed factor.

Data available from the Dryad Digital Repository: http://dx.doi.org/10.5061/dryad.m9m4f.

## Results

### Old Monkhead was more competitive than modern 92-46

**Figure 2:**
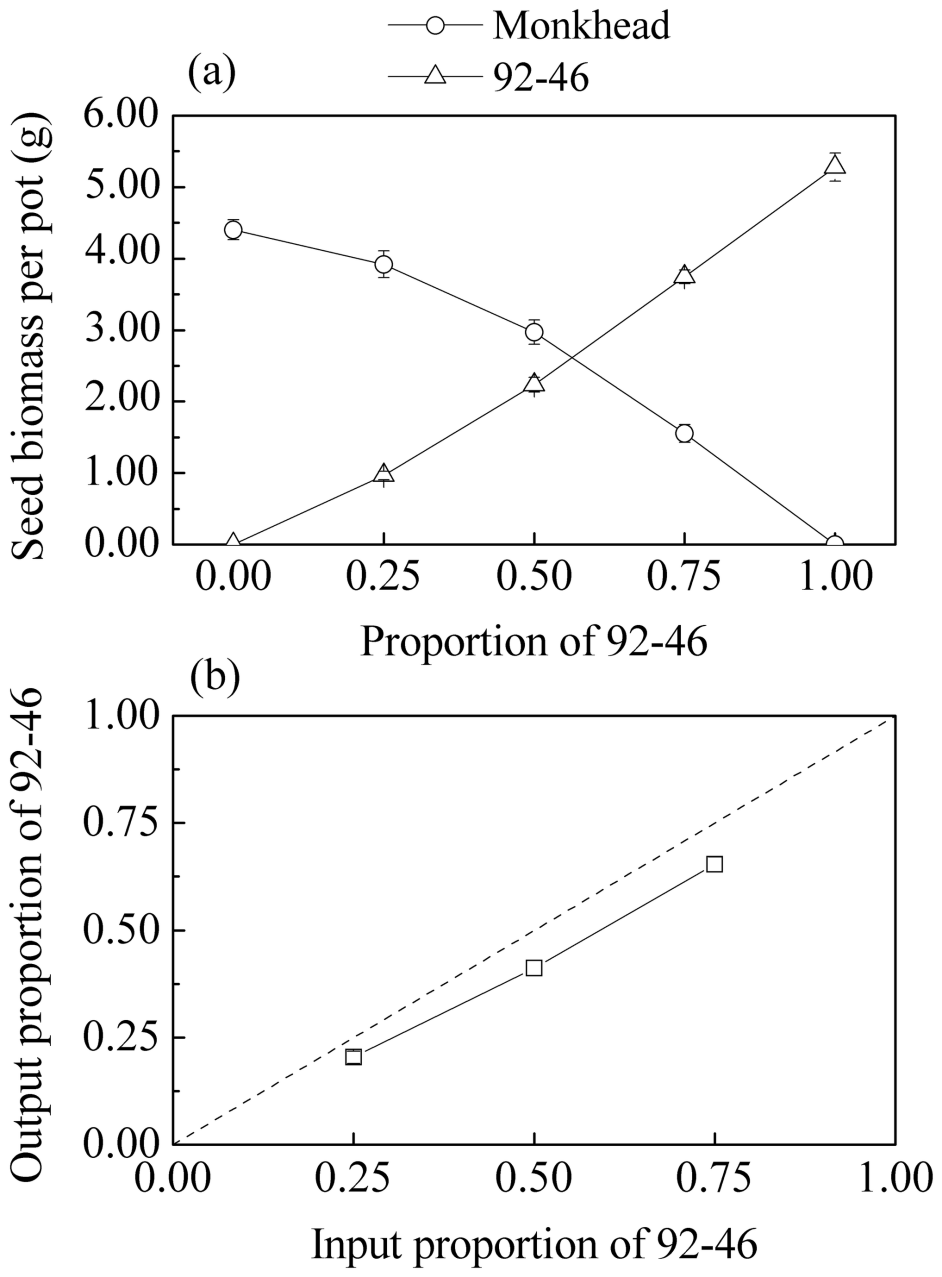
Outcome of competition between Monkhead and 92-46. (a) Seed biomass per pot of Monkhead and 92-46 along the de Wit series. Error bars denote 95% conﬁdence intervals for the mean. (b) The input–output ratio diagram, in which the output proportion of 92-46 at harvest is plotted against the input proportion of 92-46 at sowing. The dotted line is the 45° equilibrium line on which the output proportion equals the input proportion. Error bars denote 95% conﬁdence intervals for the mean.

Comparing the two monocultures, Monkhead produced less seed biomass than 92-46 (4.40 vs. 5.28 g pot^-1^, *P* < 0.01), but in mixture 2:2, Monkhead produced more seed biomass (2.97 vs. 2.23 g pot^-1^, *P* < 0.01), implying that Monkhead had an advantage over 92-46 in seed production when growing together (Figure 2a).

In the input–output ratio diagram (Figure 2b), output proportions of 92-46 were significantly (*P* < 0.01) lower than corresponding input proportions in all three mixtures: 0.20 (mixture 1:3), 0.41 (mixture 2:2), and 0.65 (mixture 3:1), suggesting that old Monkhead outperformed 92-46 in producing more seeds when growing together.

If seed biomass is used in the calculation of output proportions instead of seed number, we still got similar significantly smaller output proportions, although in the case of mixture 3:1 it was only marginally significant (*P* = 0.059). The reason is that 92-46 tended to produce larger seed without increasing seed number under more favorable growing conditions, while the seed size of Monkhead tended to remain constant.

### Old Monkhead produced more roots but fewer seeds than modern 92-46

Examining the two monocultures, we found that the root system size of Monkhead was 2.5 times larger in biomass than that of 92-46 (0.52 vs. 0.15 g plant^-1^, *P* < 0.01), revealing a higher degree of root redundancy. Seed biomass of Monkhead was lower than 92-46 (1.10 vs. 1.32 g plant^-1^, *P* < 0.01), although its total biomass was higher (3.74 vs. 2.71 g plant^-1^, *P* < 0.01).

### Modern 92-46 increased root biomass per plant as its frequency in mixtures decreased

**Figure 3:**
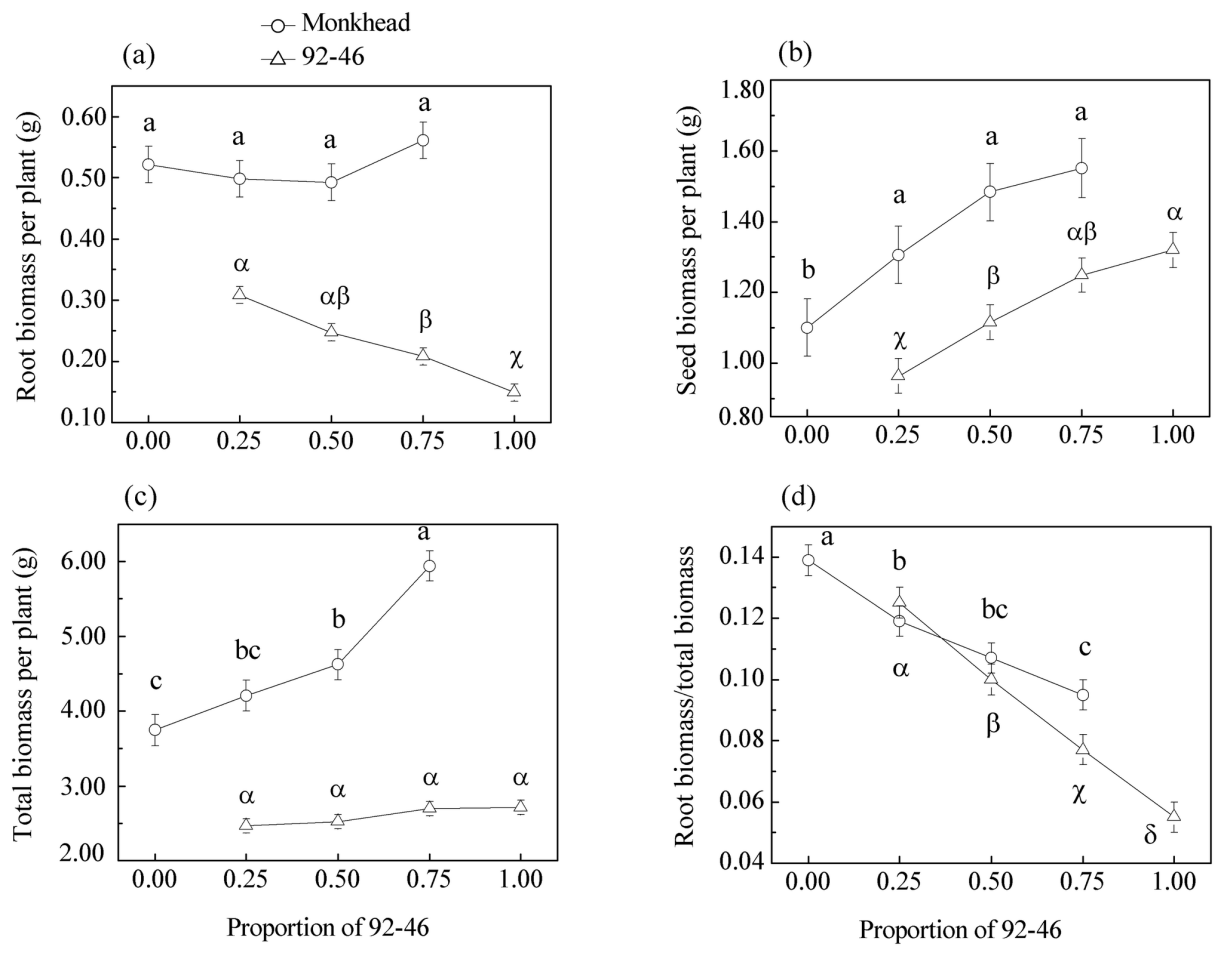
Root biomass per plant (a), seed biomass per plant (b), total biomass per plant (c), and root allocation (d) of Monkhead and 92-46 along the de Wit series. Error bars denote 95% conﬁdence intervals for the mean. We used different English letters to indicate signiﬁcant difference between different mixtures for Monkhead, and Latin letters for 92-46 (*S-N-K* test was used if variances were homogeneous; otherwise, Tamhane *t*-test was used).

Root biomass per plant of Monkhead did not change along the de Wit series (*P* = 0.35), but for 92-46 it increased significantly as its frequency in mixtures decreased (*P* < 0.01) (Figure 3a). With increasing proportion of 92-46 in mixtures, seed biomass per plant increased for both cultivars (Figure 3b), but total biomass increased only for Monkhead (Figure 3c). Two cultivars had contrasting responses to intra-cultivar versus inter-cultivar competition: Monkhead increased, whereas 92-46 decreased, root allocation (i.e., root biomass/total individual biomass) as their respective frequency in mixture increased (Figure 3d).

## Discussion

### Modern cultivar had less root redundancy and higher yield in water-limited environments

Using the de Wit replacement series method we showed that old Monkhead was more competitive but less yielding than modern 92-46 (Figure 2), thus providing further support to the hypothesis of Donald’s [4] weakly competitive ideotype or Zhang et al.’s [5] growth redundancy. As a local landrace in the semi-arid regions of China, Monkhead presumably had experienced drought conditions for long enough, hence likely having lots of opportunities to improve individual competitiveness through ‘redundant’ investment in root growth. The magnitude of root redundancy could be substantial, as in our pot experiment the landrace cultivar produced much more roots than the modern cultivar (0.52 vs. 0.15 g plant^-1^). This implies that there is still considerable room to enhance crop yield under drought conditions through plant breeding. It is worth noting that the spatial separation of roots through bagging in our experimental design limits strong competitors from invading the territories of neighboring plants, which if anything would predispose competition to be more symmetric. As explicitly modeled in Zhang et al. [5], the degree of competitive asymmetry plays an important role in determining the potential of growth redundancy.

There have been quite a few works testing Donald’s idea, neatly summarized in a recent book on Darwinian Agriculture [3]. However, most of them have focused on crop height in predominantly light-limiting environments, with the corresponding relevant root system traits in water-deficit environments being relatively under-explored. Therefore, the present study complements the previous efforts towards verifying Donald’s advocacy that ideal crop plants should be weak competitors to maximize yield. In this context, we note that the root systems of ‘green revolution’ wheat genotypes were smaller than earlier genotypes and landraces [14], although it was not seen this way. Waines and Ehdaie [14] considered smaller root systems beneficial to crop yield in irrigated and well-fertilized conditions, but we emphasize it is precisely underground competition that leads to root redundancy. In general, excessive or redundant growth in certain resource-foraging structures is adaptive in a competitive setting, but detrimental to whole-crop performance. This evolutionary trade-off between individual competitiveness and crop yield potential would have left considerable room for genetic improvement by humans [3].

Note that all previous experimental tests of the game theoretical prediction under conditions of below-ground competition have focused exclusively on the plastic behavior of roots grown alone vs. with competitors [8]. By contrast, we here consider root allocation as a genetically fixed trait for each cultivar. However, one limitation of the present work is that our sample size is only one for each group of modern versus old cultivars. Surely any conclusions based on such a small sample size can only be tentative. For example, the old cultivar, Monkhead, is awnless and this attribute has been suggested to play a role in yield performance [cf. 4].

### Modern 92-46 is more cooperative than old Monkhead not only by means of lowered investment in, but also proportionally less allocation to, root system growth

It is well known that it is notoriously hard to separate roots of individual plants from those of other conspecifics. But one great advantage of our experimental design through bagging is that we can easily determine the root biomass of each cultivar in mixtures. In particular, we found modern cultivar 92-46 decreased both its root biomass and relative allocation to roots (i.e. root biomass/total individual biomass) as its frequency in mixtures increased. However, old Monkhead did not change its root biomass (Figure 3a) and even increase root allocation under intra-cultivar competition (Figure 3d). Why do they respond to intra- versus inter-cultivar competition in such a contrasting way?

One possibility is that Monkhead, as a superior competitor, is able to decrease underground resources (water and/or nutrients) to lower levels when they prevail in mixed cultures, and both Monkhead and 92-46 responded to this increased scarcity of resources by investing more in root growth, as most clearly supported by the root allocation pattern (Figure 3d). However, only 92-46 increased root biomass in absolute terms with increased competitive pressure, whereas the root biomass of Monkhead remained invariable at individual level across different mixtures (Figure 3a). Furthermore, in a glasshouse experiment where a single plant was grown in a pot and subject to different water-nutrient availabilities, we did not find a similar increase in root biomass with environmental stress for modern 92-46, although root allocation increased for both cultivars (unpublished data). So at least for 92-46, resource scarcity appears not to be the sole reason behind the increased root growth when competing relatively more with inter-cultivar neighbors.

An alternative, and not mutually exclusive, explanation is that modern 92-46 is somehow able to distinguish between intra- and inter-cultivar neighbors [19]. Plants are generally believed to sense the external environment and respond to fluctuations in resource availability by applying physiological controls that alter development. A well-known example of such a sensing mechanism is the phytochrome system that provides information about the presence of competing neighbors [20]. However, there is also accumulating evidence that communication among plants may also occur as a result of root recognition, possibly mediated through root exudates [e.g. 7,19,21,22]. Modern cultivar increased root allocation in the presence of inter-cultivar neighbors, indicating the capacity of neighbor-identity recognition at the root level [7,19,21,22]. Or put in other way, modern cultivar plants become more cooperative by refraining from growing more roots when they compete with each other. But landraces and old cultivars have long been selected to maximize competitive ability, as explained above, and hence they are not expected to develop the same ability of self-restraint. So, it is quite understandable that old Monkhead plants don’t decrease either their root biomass or root allocation in pure stands. Old Monkhead may simply respond to resource depletion instead of neighbor identity. In terms of game theory, Monkhead could be regarded as behaving like an ESS, a strategy that does well against copies of itself, whereas modern 92-46 sacrifices its competitiveness for higher yield by growing less roots, especially when competing against like neighbors. In a similar vein, it has been argued that reduced elongation in response to crowding (i.e., reduced phenotypic plasticity) might also be a group-selected trait in wheat [6].

Finally, we present a cautionary note that the pattern of root responses to neighbor identity may be artificial. The nylon bags we used may be insufficient to entirely prevent roots of different individuals from physically penetrating through them, thus superficially enlarging the root biomass of modern cultivar plants in mixtures. However, we don’t think this scenario likely, for the following reasons. First, roots at the top which made up most of root biomass were much thicker than the pore size (150 µm) and apparently could not penetrate through nylon bags. Therefore, if anything, the effects must be very small. Secondly, even if roots thinner than 150 µm could possibly succeed in penetrating through the bags, they would have to overcome two layers of nylon net, an even more unfeasible task. And finally, we suspect only part of the successfully colonizing roots could continue to grow in the alien environment of neighboring bags, because the invading roots are “bottlenecked” to the rest parts of the root system and the associated shoots.

## Conclusions

In drought areas, natural selection through competition inevitably results in root redundancy, often of considerable magnitude, as manifested in the comparison between local landrace Monkhead and modern cultivar 92-46. Following the Donald’s lead, agronomists have become increasingly realized that the main improvements of cereal crop yields are actually based on the trade-offs between individual competitiveness and whole-crop productivity [3,6]. Furthermore, we found modern 92-46 restrained its root growth when its frequency increased in mixtures, whereas old Monkhead did not possess the same capacity. We speculate that 92-46 may have gained the ability to recognize neighbors and cooperated with its own kind, likely through root exudates Therefore, 92-46 may show a trend from competitiveness to cooperation not only in terms of lower investment in competitive ability but also of more plastic self-restraining response to intra-cultivar competition. Apparently more work is needed to confirm this proposal.

## References

[1] Tilman D, Balzer C, Hill J, Befort BL (2011) Global food demand and the sustainable intensification of agriculture. Proc Natl Acad Sci U S A 108: 20260-20264. doi:10.1073/pnas.1116437108. PubMed: 22106295. 22106295 10.1073/pnas.1116437108 PMC3250154

[2] Evans LT (1993) Crop Evolution, Adaptation, and Yield. Cambridge: Cambridge University Press. 500pp.

[3] Denison RF (2012) Darwinian Agriculture: How Understanding Evolution Can Improve Agriculture. Princeton University Press.

[4] Donald CM (1968) The breeding of crop ideotypes. Euphytica: 1968: 385-403.

[5] Zhang DY, Sun GJ, Jiang XH (1999) Donald’s ideotype and growth redundancy: A game theoretical analysis. Field Crops Res: 179-187.

[6] Weiner J, Andersen SB, Wille WKM, Griepentrog HW, Olsen JM (2010) Evolutionary Agroecology: the potential for cooperative, high density, weed-suppressing cereals. Evol Applications 3: 473-479. doi:10.1111/j.1752-4571.2010.00144.x. 10.1111/j.1752-4571.2010.00144.x PMC3352502 25567940

[7] Gersani M, Brown JS, O’Brien EE, Maina GM, Abramsky Z (2001) Tragedy of the commons as a result of root competition. J Ecol 89: 660-669. doi:10.1046/j.0022-0477.2001.00609.x.

[8] McNickle GG, Dybzinski R (2013) Game theory and plant ecology. Ecol Lett 16: 545-555. doi:10.1111/ele.12071. PubMed: 23316756. 23316756 10.1111/ele.12071

[9] Hardin G (1968) The tragedy of the commons. Science 162: 1243-1248. doi:10.1126/science.162.3859.1243. PubMed: 17756331. 5699198

[10] Marshall DR (1991) Alternative approaches and perspectives in breeding for higher yields. Field Crops Res 26: 171-190. doi:10.1016/0378-4290(91)90034-S.

[11] Jennings PR (1964) Plant type as a rice breeding objective. Crop Sci 4: 13-15. doi:10.2135/cropsci1964.0011183X000400010005x.

[12] Fang Y, Liu L, Xu BC, Li FM (2011) The relationship between competitive ability and yield stability in an old and a modern winter wheat cultivar. Plant Soil 347: 7-23. doi:10.1007/s11104-011-0780-4.

[13] Siddique KHM, Belford RK, Tennant D (1990) Root: shoot ratios of old and modern, tall and semi-dwarf wheats in a Mediterranean environment. Plant Soil 121: 89-98. doi:10.1007/BF00013101.

[14] Waines JG, Ehdaie B (2007) Domestication and crop physiology: roots of green-revolution wheat. Ann Bot 100: 991-998. doi:10.1093/aob/mcm180. PubMed: 17940075. 17940075 10.1093/aob/mcm180 PMC2759207

[15] Zhang R, Zhang DY (2000) A comparative study on root redundancy in spring wheat varieties released in different years in semi-arid areas Acta Phytoecologica Sinica 24: 298-303 (in Chinese with English abstract)

[16] Zhang R, Zhang DY, Yuan ZB (1999) A study of the relationship between competitive ability and productive performance of spring wheat in semi-arid regions of loess plateau. Acta Phytoecologica Sin 23: 205-210 (in Chinese with English abstract)

[17] de Wit CT (1960) On competition. Versl landbouwk underz 66: 1-82.

[18] Harper JL (1977) The Population Biology of Plants. London: Academic Press.

[19] Dudley SA, File AL (2007) Kin recognition in an annual plant. Biol Lett 3: 435-438. doi:10.1098/rsbl.2007.0232. PubMed: 17567552. 17567552 10.1098/rsbl.2007.0232 PMC2104794

[20] Smith H (2000) Phytochromes and light signal perception by plants - an emerging synthesis. Nature 407: 585-591. doi:10.1038/35036500. PubMed: 11034200. 11034200 10.1038/35036500

[21] Chen BJ, During HJ, Anten NP (2012) Detect thy neighbor: Identity recognition at the root level in plants. Plant Sci 195: 157-167. doi:10.1016/j.plantsci.2012.07.006. PubMed: 22921010. 22921010 10.1016/j.plantsci.2012.07.006

[22] Fang S, Clark RT, Zheng Y, Iyer-Pascuzzi AS, Weitz JS et al. (2013) Genotypic recognition and spatial responses by rice roots. Proc Natl Acad Sci U S A 110: 2670-2675. doi:10.1073/pnas.1222821110. PubMed: 23362379. 23362379 10.1073/pnas.1222821110 PMC3574932

